# Proteomic Research of Extracellular Vesicles in Clinical Biofluid

**DOI:** 10.3390/proteomes11020018

**Published:** 2023-05-06

**Authors:** Shipan Fan, Ansgar Poetsch

**Affiliations:** 1 School of Basic Medical Sciences, Nanchang University, Nanchang 330021, China; fspan@email.ncu.edu.cn; 2 Queen Mary School, Medical College, Nanchang University, Nanchang 330021, China

**Keywords:** extracellular vesicles, proteomics, clinical application, body fluids

## Abstract

Extracellular vesicles (EVs), the lipid bilayer membranous structures of particles, are produced and released from almost all cells, including eukaryotes and prokaryotes. The versatility of EVs has been investigated in various pathologies, including development, coagulation, inflammation, immune response modulation, and cell–cell communication. Proteomics technologies have revolutionized EV studies by enabling high-throughput analysis of their biomolecules to deliver comprehensive identification and quantification with rich structural information (PTMs, proteoforms). Extensive research has highlighted variations in EV cargo depending on vesicle size, origin, disease, and other features. This fact has sparked activities to use EVs for diagnosis and treatment to ultimately achieve clinical translation with recent endeavors summarized and critically reviewed in this publication. Notably, successful application and translation require a constant improvement of methods for sample preparation and analysis and their standardization, both of which are areas of active research. This review summarizes the characteristics, isolation, and identification approaches for EVs and the recent advances in EVs for clinical biofluid analysis to gain novel knowledge by employing proteomics. In addition, the current and predicted future challenges and technical barriers are also reviewed and discussed.

## 1. Introduction

The general term “extracellular vesicles (EVs)” refers to particles (40 nm–10 µm) with lipid bilayer membranes, which are produced by and released from almost all cells, including eukaryotes and prokaryotes. The versatile (patho-)physiological role of EVs has been investigated in development, coagulation, inflammation, immune response modulation, and disease by cell–cell communication [1]. The first hint of particulate fractions (EVs) with coagulant activity was obtained after isolating blood-coagulating proteins from plasma by high-speed centrifugation [2] where the lipid-rich particles were described as platelet-derived microstructures of varied diameters and densities and were termed “platelet dust” [3]. Initially, by investigating reticulocytes, EVs were thought to discard garbage from cells and named exosomes [4,5]. Later, Raposo et al. [6] found exosomes derived from human and murine B lymphocytes to mediate antigen presentation. Furthermore, Zitvogel et al. [7] observed that exosomes secreted from dendritic cells suppress tumor growth, which implied that exosomes partake in intercellular communication. Valadi et al. [8] first identified exosomes containing both mRNA and microRNA, which can be transferred to recipient cells and trigger signal transduction.

**Figure 1:**
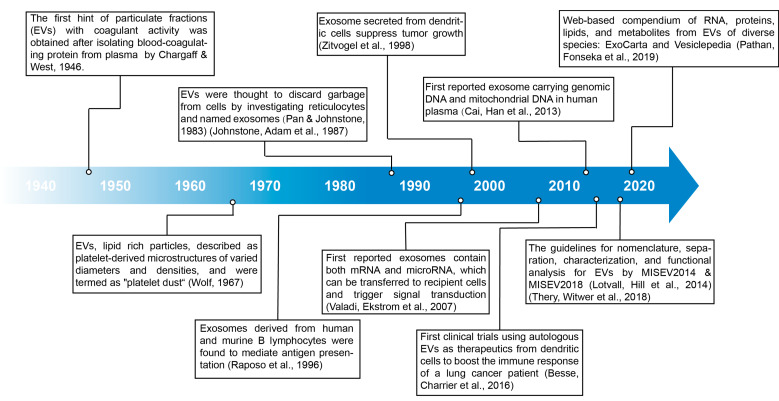
Timeline of EV research [2,3,4,5,6,7,8,9,10,11,12,21].

Cai et al. first reported exosomes carrying genomic DNA and mitochondrial DNA in human plasma [9]; in the same year, the Nobel Prize was awarded for the discovery of vesicle trafficking, then Besse et al. [10] gave impetus to the first clinical trials and used autologous EVs as therapeutics from dendritic cells to boost the immune response of a lung cancer patient. Incited by the tremendous medical prospects, the scientific community has enthusiastically produced a wealth of studies and addressed the need for guidelines and standardization. Worth noting are the efforts by Lotvall and Thery et al. who updated guidelines of nomenclature, separation, characterization, and functional analysis for EVs with minimal information for studies of EVs, MISEV2014 and MISEV2018, to generate references to make data reliable and reproducible between labs [11,12] (Figure 1).

This review addresses EV features, common and emerging methods in EV sample preparation, and their potential for diagnosis and therapy with a focus on contributions made by proteomics. In addition, the current and future challenges and barriers are also reviewed and discussed.

## 2. The Features of EVs

EVs are produced and released by cells from all living organisms; further classification of EVs is based on size, biogenesis, and composition [12] with the diversity of EVs expanding continuously [13]. According to biological function and features, EVs are mainly divided into three categories: apoptotic bodies, microvesicles, and exosomes. It is evident from Table 1 that these three classes of EVs substantially overlap in their physicochemical features. This heterogeneity poses a great challenge for purification from biological samples. Apoptotic bodies are secreted merely by direct budding from the plasma membrane of dying cells and enclosed with the fragments of the cellular components [14] while microvesicles originate via shedding of the plasma membrane. The outward budding of microvesicles is controlled by intracellular Ca^2+^ levels, and they consist of an intracellular set of proteins and trapped materials that contribute to cellular communication, signal transduction, or metabolism of protein and nucleic acid [15,16]. Notably, exosomes generated from various cells during the inward budding process of endocytosis: The first invagination of the plasma membrane forms the early endosome before a second invagination gives rise to develop intraluminal vesicles (ILVs) within the late endosome, known as multivesicular bodies (MVBs). Next, the limiting membrane of the MVBs fuses with the plasma membrane, then releases ILVs into the extracellular milieu, now named exosomes; their cargoes are cytosolic proteins and lipids, as well as trapped molecules, such as metabolites and nucleic acids, which specifically mirror the physiology of their cellular origin during their biogenesis [17,18]. To promote comprehension of EVs’ complexity, ExoCarta [19] and Vesiclepedia [20] were launched, two continuously updated web-based databases incorporating RNA, proteins, lipids, and metabolites in EVs of diverse species [21] (Figure 1) . According to the published literature, the exosome is the most well-studied type of all EVs, sorting proteins and other materials into recipient cells and triggering complex intracellular pathways to regulate various processes, including development, coagulation, inflammation, immune response modulation, and disease by cell–cell communication [1,22,23,24,25]. Known for their ability to package and convey cargo, microvesicles play a role in the pathophysiological process of humans as well. Given their nanoscale size and natural lipid bilayer abound with adhesive proteins to fuse with the plasma membrane of recipient cells, EVs (which primarily refer to exosomes and small microvesicles) prospectively represent an attractive source of a diagnostic biomarker for disease or as drug delivery vehicles [26,27].

## 3. An Overview of the EV Biology in Disease Context

**Figure 2:**
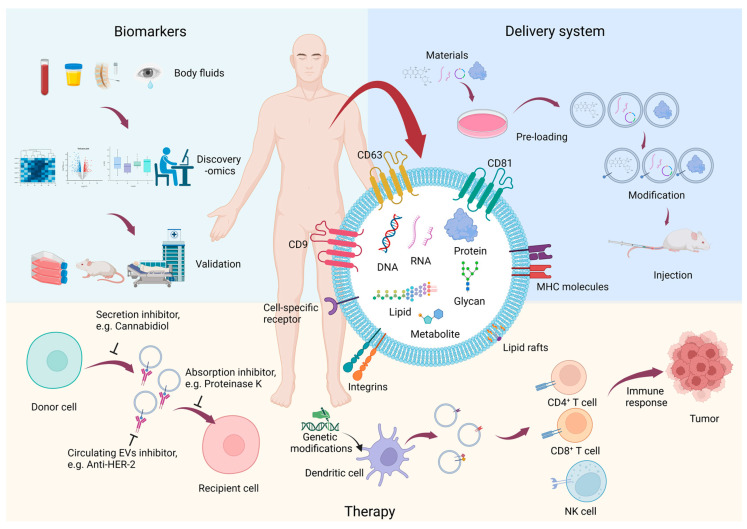
The application of EVs derived from various body fluids (created with BioRender.com).

The intracellular cargoes of EVs are a rich source of disease-associated molecules, which are considered to have great potential as a noninvasive source of biomarkers in various models of health and disease, which can be readily isolated from a wide range of almost all physiological fluids in the body, such as plasma, saliva, cerebrospinal fluid, amniotic fluid, breast milk, urine, and so forth [34]. Emerging clinical applications are engineered vesicles as a promising drug delivery tool, especially in central nervous system diseases, to cross the blood–brain barrier thanks to their nanoparticle size and the ability to transfer cargo to distant sites throughout the body by delivering it in a soluble format and concentrated status [35]. For instance, Han et al. [36] utilized a vibrating mesh nebulizer to deliver small EVs loaded with small RNAs to alleviate lung injury in mice, demonstrating the therapeutic potential. Many publications [37,38] demonstrated that EVs derived from tumors generate a premetastatic niche in distant organs and modulate immunity, thereby promoting tumor metastasis and immune escape in the tumor microenvironment by regulating subsequent signal transduction in recipient cells, illustrating the potential therapeutic value of EVs. Moreover, increasing reports [39] present EVs as a pivotal mediator in host innate immune responses, and Xiong et al. [40] exploited EVs from manipulated dendritic cells to generate a cell-free anticancer vaccine to inhibit tumor growth and enhance survival rate (Figure 2). Subsequently, we will discuss the recent progress of EVs research in cancer progression, immune response modulation, neurodegenerative disease, and viral infection via the proteomic tool.

### 3.1. Cancer Progression

After decades of studying EVs, it has been established that biological fluids from cancer patients contain more secreted EVs because of intensified cell-to-cell communication, which is considered an essential factor for tumor progression and therapeutic targeting [41,42]. Chang et al. [43] found an EV protein signature (six proteins) derived from the serum of colorectal cancer patients where APOF and CFB are linked to clathrin-mediated endocytosis signaling, and the complement system is considered crucial for the development of tumorigenesis. Matthiesen et al. [44] collected EVs from plasma and performed proteomic profiles to distinguish diffuse large B cell lymphoma cancer patients and proposed the use of EV protein indicators to predict prospective survival. By virtue of quantitative proteomics and further verification through ELISA and immunoblot, Hou et al. [45] found that Stratifin, a member of the 14-3-3 protein family generated from the serum EVs of colorectal cancer patients, is a biomarker to predict prognosis. In addition, EVs directly extracted from tissue samples exhibit excellent tissue specificity and an intimate relationship to the microenvironment. Zhang et al. [46] utilized the specific binding between TiO_2_ and phosphate groups to isolate EVs and conduct a proteomic analysis of about 11 biomarkers for hepatocellular carcinoma. EVs isolated from tumor cell lines are purer and more homogenous than from liquid biopsy and were used to investigate the regulation mechanism of proliferation, invasion, and metastatic dissemination [47,48,49,50,51].

### 3.2. Immune Response Modulation

EVs have been found to deliver protein, lipids, and nucleic acid cargo to play key roles in the immune response modulation system and trigger activating and suppressive functions via intercellular communication [52,53]. EVs are regarded as a useful and prospective therapeutic tool to enhance antitumor immunity and improve the outcome of cancer treatment. Gargiulo et al. [54] isolated EVs to observe and analyze surface protein expression in immune regulation and then displayed time-dependent changes in the immune response and metabolism pathway after CD8^+^T cells were treated with EVs derived from a leukemia microenvironment to demonstrate they were remodeling the immune microenvironment of the chronic lymphocytic leukemia mouse model. Human milk not only supports the growth and development of newborns but also contains EVs to benefit the health of infants by influencing the immune system [55]. In a reproduction study, Jena et al. [56] compared the proteome of EVs originating from semen and suggested GDF-15 and C3 related to impaired immune response modulation in recurrent pregnancy loss patients. Finamore et al. [57] evaluated the differential expressed protein in EVs derived from saliva between primary Sjögren’s syndrome patients and healthy donors, which indicated the protein–protein interaction network involved in the innate immune response process. Gerwing et al. [58] separated EVs derived from a 4T1 breast cancer cell and compared the protein inventory to a healthy group, then injected tumor EVs into healthy mice to show the alteration of immune cell composition in distant metastatic organs.

### 3.3. Neurodegenerative Disease

The fact that EVs can cross the blood–brain barrier not only exhibits the potential for drug delivery research in the central nervous system but also yields valuable information on neurodegenerative disease [59]. The three main neurodegenerative diseases are Alzheimer’s disease (AD), Parkinson’s disease (PD), and amyotrophic lateral sclerosis (ALS). You et al. [60] utilized four neural cell types, including excitatory neurons, astrocytes, microglia-like cells, and oligodendrocyte-like cells, to isolate their EVs, respectively, and identified protein markers for specific cell types by analyzing their proteome. After protein co-expression network analysis in human brain-derived EVs, they found EVs derived from astrocytes are the most significant enrichment module and highlighted the potential pathogenesis mechanism in AD. Additionally, promising and novel AD biomarkers of EVs derived from various sources were discovered [61,62,63,64,65,66,67], and researchers compared the proteomic landscape following the knockout or overexpression of important genes to understand AD pathology [68,69]. Jewett et al. [70] revealed the dysregulated protein of EVs isolated from the Gba1b mutant (GBA deficiency) Drosophila model to promote abnormal protein aggregation in neurons of PD. ALS is a heterogeneous, multifactorial, and fatal neurodegenerative disease; Vassileff et al. [71] identified 16 proteins associated with ALS from the proteome of EVs separated from the motor cortex and demonstrated their potential to indicate ALS. Thompson et al. [72] detected the protein alteration of EVs derived from CSF of ALS patients and regarded this as a potential biomarker, while Sjoqvist et al. [73] found no differentially expressed protein in EVs derived from CSF by using a ultra-sensitive proximity extension assay. Thus, further research is necessary to establish robust biomarkers for early diagnosis and treatment.

### 3.4. Viral Infection

EVs can either accelerate the infection of neighboring cells via the transport of infectious viral particles or induce the antiviral response to assist the host cell in curbing the infection [74,75]. For Epstein–Barr virus (EBV), which is associated with many diseases, Xie et al. [76] applied proteomic analysis in exosomes derived from the plasma of EBV-hemophagocytic lymphohistiocytosis patients and listed key proteins for diagnostic biomarkers. Ito et al. [77] found that integrin αLβ2 and FGF2 mediate the emergence of tumor-associated macrophages from surrounding phagocytes, which were induced by the specific EV subtype (phosphatidylserine-exposing exosomes). Human immunodeficiency virus (HIV) infection leads to acquired immunodeficiency syndrome and other disorders [78]; Falasca et al. [79] compared the protein contents of EVs derived from endothelial cells, leukocytes, and platelets from HIV patients with healthy volunteers and suggested EVs upregulated chronic inflammation to facilitate viral replication after HIV infection through γIFN, IL1α, NF-κB, and JAK/STAT3 signaling pathways. At present, COVID-19 still influences the world as a pandemic infectious disease; Pesce et al. [80] isolated EVs from the plasma of mild and severe COVID-19 patients followed up by proteomics with healthy donors as the control group and observed both mild and severe case-derived EVs to be involved in the upregulation of the immune response for SARS-CoV-2, but differences in the immunomodulatory effects in the activation of immune cells (CD4^+^T-cell) and acute inflammation, respectively, associated with different protein signatures in EVs. Barberis et al. [81] studied the pathogenesis of COVID-19 based on the proteome of plasma-derived EVs and found enrichment of the immune response and inflammation enabling coagulation and complement pathways.

## 4. Methods of EV Isolation

### 4.1. Conventional Approaches for the Isolation of EVs

Many methods have been developed by researchers to enrich EVs based on their physical (sedimentation coefficient, size, and density), biochemical, and affinity properties [82]. Differential ultracentrifugation based on the different sedimentation rates of particles with physical characterizations is the primary method, the gold standard, to isolate EVs and is widely used among labs despite its limitations, such as low sample recovery rate, low throughput, potential damage to EVs, and contamination from soluble proteins. Large-volume samples are especially suitable for ultrafiltration, which concentrate EVs by a certain molecular weight under low-speed centrifugation but are prone to detrimental membrane clogging effects and contamination with unspecific proteins. To improve ultrafiltration performance, a tangential flow filtration (TFF) system was developed to reduce the formation of filter cake and enhance the efficiency of filter membranes via laminar flow. Size-exclusion chromatography (SEC) utilizes resins, a porous stationary phase, in which the elution times of various materials are different; it is low-cost and quick, while the capacity is limited for sample volume and does not reach complete purity. According to the densities of EVs being lower than proteins, density gradient ultracentrifugation separates EVs effectively but more laboriously than differential centrifugation. Common pre-treatment is filtering through a 0.22 µm filter to remove cell debris, lysosomes, etc. EVs also can be precipitated with polymers, but the method is prone to contamination with aggregates from untargeted proteins. Affinity-based methods that extract EVs via the affinity interaction of surface markers are often combined with chromatography and provide high purity by exploiting the biological signature of specific subpopulations [83]. Examples are immunoaffinity (e.g., Veneceremin binds HSPs at the surface of EVs specifically), lectin-glycoproteins affinity, and lipid affinity. Moreover, peptide [84] and aptamer-based [85] affinity materials were engineered and developed for the isolation and recognition of EVs.

In addition, the strategy of combining the above methods to isolate EVs from various samples has been demonstrated superior to a single method yet may pertain to sample loss and a laborious workflow [86,87]. There is no ideal method for EV separation according to the ISEV (International Society for Extracellular Vesicles), and the choice of techniques depends on the downstream application and scientific question in conjunction with desired recovery and specificity (Table 2). Despite the intense development of novel methods lately, the most widely used and most accessible method remains differential ultracentrifugation. Until now, no comprehensive experimental comparison of method performance for proteomics has been made.

A strong impact of the sample preparation method on proteome results has been appreciated in various studies: Askeland et al. [88] compared ultracentrifugation, SEC, and a precipitation kit in plasma-derived EV biomarker studies through MS analysis and considered ultracentrifugation and SEC as suitable approaches for large and small EVs, respectively. Tuner et al. [89] evaluated the combination of ultracentrifugation as well as only ultracentrifugation or SEC to enrich plasma EVs, and they suggested ultracentrifugation with subsequent SEC was the best method for proteome profiling. Mussack et al. [90] used five urinary EV purification methods and found a significant method-dependent difference in protein composition. Karimi et al. [91] combined SEC with a density gradient centrifugation, which alleviated the contamination from lipoproteins and facilitated proteome analysis of plasma EVs. Tauro et al. [92] noted that the isolation of exosomes from a tumor cell line conditional medium by immunoaffinity capture outperforms ultracentrifugation and density-based separation for proteomics. Wang et al. [93] extracted serum exosomes through a magnetic affinity separation nanoplatform, which outperformed current ultracentrifugation for downstream proteomic analysis. Huang et al. [94] compared four methods (ultracentrifugation, Size-exclusion chromatography, ExoQuick-TC precipitation, and ExoQuick-TC ULTRA isolation) and also presented method-dependent differences in proteomes. The latter illustrates a general challenge when pursuing purification enrichment of the desired type of exosomes and avoiding its unintended depletion due to insufficient knowledge of their biochemical and/or biophysical properties.

### 4.2. Advanced Approaches for the Isolation of EVs

The innovations and endeavors of researchers are contributing to the development of upgraded and new techniques aimed at elevating the efficiency and purity of EV isolation from MISEV2018, the consensus of the ISEV. There is a sharp increase in new techniques for promoting higher purity in EV isolation.

#### 4.2.1. Asymmetric Flow Field Flow Fraction, AF4

A nanoparticle detection device was established for field flow fraction (FFF), which was developed by Giddings et al. [95]. A size-based purification method with two perpendicular flows creates a force field to avoid mechanical and shear stress for EV separation to achieve a high resolution and broad size range but is limited to small sample loads and low yield. Moreover, Zhang et al. [96,97] utilized optimized AF4 and identified two EVs and a novel nanoparticle termed exomeres.

#### 4.2.2. Microfluidic-Based Technologies

An integrable module of separation and detection for EVs with a low risk of cross-contamination to simplify the complicated multiple workflows of conventional methods was advertised as a promising tool in clinical diagnosis [98]. The isolation, detection, and analysis parts are based on acoustic nanofiltration, deterministic lateral displacement, viscoelastic flow sorting, plasmonic sensing, and electrochemical sensing. Xu et al. [99] reported a ZnO-nanorods integrated (ZNI) microfluidic chip device that captured EVs onto the surface of nanomaterials via immunoaffinity and detected the fluorescent signal of Vimentin, the osteosarcoma biomarker, to increase sensitivity for distinguishing osteosarcoma and metastatic disease effectively. Lo et al. [100] refined an immune affinity-based microfluidic device, ExoChip, coated by the CD63 antibody to capture EVs derived from the blood of amyotrophic lateral sclerosis patients specifically. Sung et al. [101] described an automated and highly sensitive integrated microfluidic platform featuring a sample treatment to microRNA biomarker quantification from 20 µL plasma in ovarian cancer. Rima et al. [102] developed a novel microfluidic system to collect EVs generated from breast cancer tumor spheroids, thereby enabling analysis at the single-vesicle level. Niu et al. [103] designed a fluid nanoporous microinterface (FluidporeFace) in a microfluidic chip to enhance the isolation efficiency of EVs derived from the tumor.

#### 4.2.3. Dichotomic SEC

Guo et al. [104] described an optimized dichotomic SEC method using a CL-6B column with increased bed volumes to produce a high-level yield of EVs and a low level of contaminants without multiple fractions and pooling operations.

#### 4.2.4. Ultrafast-Isolation System, EXODUS

Chen et al. [105] developed EXODUS to purify EVs from various body fluids with outstanding efficiency via negative pressure oscillation (NPO) and double coupled harmonic oscillator (HO)-enabled membrane vibration to isolate EVs after removing contaminants (nuclear acids and proteins) to support downstream omics analysis in urine, cerebrospinal fluid, plasma, and tears [106,107,108,109] with limited throughput yet effective fractionation into three nanopore sizes (20, 100, and 200 nm).

#### 4.2.5. EV Enrichment Device, EVrich

Zhang et al. [110] designed a magnetic beads-based device using EVtrap beads, which were modified with a combination of hydrophilic and lipophilic groups that have a unique affinity (non-antibody-based) toward lipid-coated EVs to recover and purify EVs in 96-well plates, which enabled a high-throughput and automated process for microRNA, proteomics, and phospho-proteomics analysis directly with minimal hands-on time.

#### 4.2.6. Commercial Exosome Isolation Kits

Several commercial exosome isolation kits (Table 3) have been developed, such as Exo-spin™, ExoQuick™ Exosome Precipitation, Total Exosome Isolation Reagent from Invitrogen™, the PureExo^®^ Exosome Isolation Kit, the miRCURY™ Exosome Isolation Kit, the ExoSure™ Exosome Isolation Kit, the MagCapture™ Exosome Isolation Kit, the Hieff^®^ Quick Exosome Isolation Kit, the exoEasy™ Maxi Kit, the EasySep™ Extracellular Vesicle PE Positive Selection Kit, the Capturem™ Extracellular Vesicle Isolation Kit, and the ExoPure™ Isolation Kit, for different sample types.

#### 4.2.7. Others

For downstream analysis of EVs by proteomics, Buck et al. [111] developed an Azo-enabled method to extract protein and digest it rapidly during sample preparation to attain high sample throughput. Wang et al. integrated a nanoporous TiO_2_-based device to separate tumor-derived exosomes with high recovery and high specificity, distinguishable from microvesicles similar to exosomes at the size level [112].

## 5. -Omics Approaches to Study EV in Clinical Biofluid

### The Role of -Omics Methods in Clinical Applications of EVs

-Omics technologies have revolutionized studies of biological regulation and our understanding of disease mechanisms by enabling high-throughput analysis of biomolecules. Moreover, this rich information has highlighted individual variation in large-scale cohort research, paving the way to personalized medicine. Various directions of -omics methods exist: Proteomics technology is a comprehensive and powerful tool for defining potential protein functional roles. In addition, genomics, epigenomics, transcriptomics, metabolomics, lipidomics, and glycomics also partake in the bioinformation flow and present (patho-)physiological changes. -Omics data from above facilitated the determination of candidate biomarkers on different molecular levels for EVs, which diagnose disease-specific subtypes, monitor the progress of diseases, or respond to therapeutic intervention. The goal of -omics is to obtain a large amount of comprehensive information in a short time to be processed by advanced computational algorithms that preserve real biological variation by eliminating systematic experimental bias and technical variation [65,113,114,115]. Achieving this goal depends on ongoing method development for EVs to deal with general and specific challenges (Table 4).

**Figure 3:**
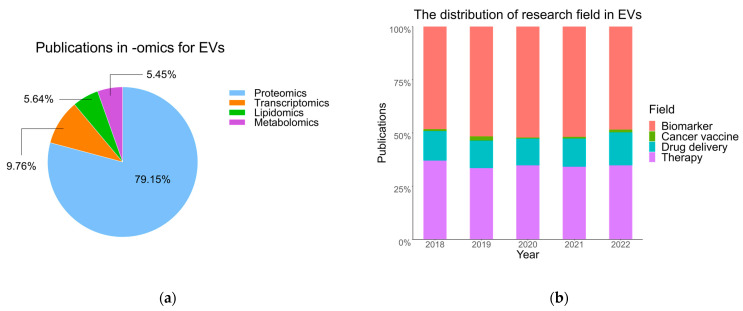
The last five years of omics research and applications for EVs (data from Web of Science, up to January 2023). (**a**) Statistical publications in omics studies for EVs. (**b**) Publications in selected clinical research areas using -omics for EVs.

Unsurprisingly, the use of -omics methods in diagnosis delivered an avalanche of biomarker candidates, showing great promise for better disease detection and treatment. Nevertheless, a stable, predictive, and interpretable biomarker inevitably undergoes a long and costly process to qualify for personalized medicine, and most biomarker candidates from -omics studies were eliminated during that development. Publications related to -omics techniques for EV studies are increasing. Most of them focus on proteomics and transcriptomics through summarizing the last five years’ literature (Figure 3a). Table 5 contains a list of candidate biomarkers originating from EVs in body fluids obtained via a proteome study and Table 6 for a transcriptome study; of note, tumor-derived non-coding RNA cargo in EVs has attracted lots of attention and attributed to the specific contents from the original cells that are faithfully and sensitively detected in EVs and were reviewed elsewhere (Ebrahimi et al. [125]). It should be mentioned that it remains to be established if annotation as “non-coding” is correct for many of these RNAs since studies [126,127] suggest the need to re-annotate as well as to newly discover protein-encoding RNAs. Proteomics is an invaluable tool to resolve this issue and may uncover new disease-relevant proteins. EVs have been further investigated in biomarker, therapy, drug delivery, and cancer vaccine fields (Figure 3b). Obviously, many biomarker candidates were identified from EVs in clinical body fluids, but very few enter clinical trials with physiological activities and ultimately obtain approval (e.g., by FDA); the road from bench to bedside is full of challenges and obstacles, such as human disease heterogeneity, limitations of surrogate disease model systems for biomarker candidates, and the difficulties in establishing a clear link between molecular indicators and disease pathology with high sensitivity and specificity [128]. Hope is given by accumulating clinical trials applied to EVs summarized by Lai et al. [121], which involves cancer (main cases), cardiovascular disease, infectious disease (COVID-19), neurodegenerative disease, and others. To date (Dec. 23rd, 2022), there are 112 ongoing clinical trials about EVs in diagnosis and therapy, including early phase 1 (9 cases), phase 1 (35 cases), phase 1/2 (21 cases), phase 2 (29 cases), phase 2/3 (5 cases), phase 3 (5 cases), and phase 4 (8 cases) [129]. ExoDxLung (ALK) is the world’s first plasma-based diagnostic enabling real-time detection of EML4-ALK mutations in non-small cell lung cancer patients, launched by Exosome Diagnostics in 2016 [130]. Although there is no licensed EV therapeutic product so far, increasing clinical trials are applied EVs in human diseases, such as EXOFLO derived from human bone marrow mesenchymal stromal cells to alleviate the moderate-to-severe acute respiratory distress syndrome of COVID-19 patients (NCT05354141) [131]. Therefore, the global market displays great enthusiasm for EVs diagnostics and therapeutics. A market report by BBC Research predicted global investment in EVs with a compound annual growth rate (CAGR) of 41.3% for the period of 2021–2026 in the diagnostics field and 38.6% in the therapeutic field [132] with a large fraction of EV research concentrated on cancer [132,133]. Moreover, Vesigen Therapeutics raised $28.5 million for the use of microvesicles in drug delivery therapy due to its benefit of carrying greater payloads than exosomes and being more readily produced at large scale [134].

There are plenty of proteome studies on the essential components of EVs in biofluids, mostly blood and urine, to identify candidate biomarkers. Proteomics is a highly promising tool for EVs and is able to classify tumor types and establish signature proteins and robust biomarkers. Hoshino et al. [135] conducted proteomic profiles of EVs from 426 human cancer samples to identify and classify uncertain primary tumor subtypes illustrating that EVs from body fluids possess a potential value to improve the remedial outcome of lethal cancer. In addition, EVs possess extraordinary features ideal for proteomics using mass spectrometry (MS) [136]: relatively low complexity, enrichment in low abundance molecules, a conserved set of common proteins that are vital for vesicle biogenesis, structure, trafficking, and the presence of specifical proteins from the (pathological) cell type they originated. Proteomics gives access to post-translationally modified (PTM) proteins that were altered in function, physicochemical properties, and cellular pathogenesis through chemical modification after translation, including cleavage of precursors, formation of disulfide bonds, covalent attachment or removal of low-molecular-weight groups, and so forth. Glycosylation, phosphorylation, ubiquitination, SUMOylation, acetylation, and S-nitrosylation have been identified and studied in EVs [137]. For example, immobilized metal affinity chromatography (IMAC) is a common method to enrich phosphopeptides with robust interaction between phosphate groups and metal ions, and hydrophilic interaction chromatography (HILIC) is a favorable method to concentrate glycopeptides due to excellent enrichment efficiency and unbiased binding; Zheng et al. [138] fabricated the above and developed a core–shell carbonyl-functionalized magnetic zirconium–organic framework (CFMZOF) to identify phosphopeptides and glycopeptides simultaneously in a human urine sample with high selectivity and a low detection limit. Nunez et al. [139] first defined the proteome landscape of diabetes patients’ serum-derived EVs and quantified the circulating global proteins and phosphoproteins simultaneously. More importantly, proteomics boosts the dissection of the protein signature, signaling pathways, and clinical pharmacokinetics of EVs by improving the proteome sequence coverage to better characterize their molecular cargo via identifying PTMs and proteoforms in particular pathological events with the ultimate aim of achieving clinical translation.

**Table 5:** List of potential disease biomarkers derived from EVs in human body fluids via proteome studies.

Source	Biomarker	Isolation Method/Identification Method	Screening Method/Verification Method	Disease	Ref.
Serum	COPB2↑	Filter column/WB, SEM	LC-MS/MS/WB, ELISA	COVID-19	[140]
Plasma and serum	HSP90A↑, STIP1↑, TAGLN-2↑	Ultrafiltration, differential centrifugation, density gradient centrifugation/TEM, NTA, WB, LVSEM	LC-MS/MS/WB	Adenomyosis	[141]
Plasma	PKG1↑, RALGAPA2↑, TJP2↑	Ultracentrifugation/WB	LC-MS/MS/PRM	Breast Cancer	[142]
Plasma	TSPAN1↑	Differential centrifugation, ExoQuick^®^/TEM, NTA, WB	LC-MS/MS/WB, ELISA	Colon Cancer	[143]
Serum	GCLM↓, KEL↑, APOF↑, CFB↓, PDE5A↓, ATIC↓	Size-exclusion chromatography/TEM, WB	LC-MS/MS/NA	Colon Cancer	[43]
Blood	ORM1	NA/NA	Large-scale targeted proteomics analysis/NA	Colon Cancer	[144]
Serum	Stratifin↑	Size-exclusion chromatography, exoEasy kit/TEM, NTA, WB	LC-MS/MS (TMT)/ELISA	Colon Cancer	[45]
Serum	Annexin A3↑, A4↑, and A11↑	Differential ultracentrifugation, density gradient centrifugation/NA	LC-MS/MS (SRM)/NA	Colon Cancer	[145]
Serum	TRIM3↓	ExoQuick^®^/WB, TEM, NTA,	LC-MS/MS/ELISA, WB	Gastric Cancer	[146]
Plasma	TGFβ1↑	Extracellular vesicles enrichment kit/TEM, NTA, WB	LC-MS/MS(TMT)/ELISA	Head and Neck Squamous Cell Carcinoma	[147]
Serum	AMPN↑, PIGR↑, VNN↑	Filtration, ultracentrifugation/TEM, NTA, WB	LC-MS/MS/WB	Liver Cancer	[148]
Plasma	SRGN↑, TPM3↑, THBS1↑, HUWE1↑	Density gradient flotation/TEM, NTA, WB	LC-MS/MS/WB	Lung Cancer	[149]
Serum	CD5L↑	Precipitation and magnetic-based immunoaffinity/TEM, NTA, WB, DLS	MALDI-TOF-MS/WB	Lung Cancer	[150]
Serum	CD91↑	MSIA monolith tips/NA	LC-MS/MS/ELISA	Lung Cancer	[151]
Serum	α-synuclein↑, Clusterin↑	Immunoaffinity/SEM, NTA, WB	LC-MS/MS/electrochemiluminescence	Parkinson’s Disease	[152]
Serum	Syntenin-1↑	Ultracentrifugation/NTA, EM, WB	LC-MS/MS/WB	Parkinson’s Disease	[153]
Plasma	IgG↑, IgM↑, C1q↑	Immunoaffinity/flow cytometry	LC-MS/MS/NA	Systemic Lupus Erythematosus	[154]
Plasma	G3BP↑, TGFβ1↓	Centrifugation/NA	LC-MS/MS/NA	Systemic Lupus Erythematosus	[155]
Urine	Hsp 90↑, syndecan-1↑, MARCKS↑, ZO-2↑	Gradient density ultracentrifugation, differential ultracentrifugation/TEM, NTA, WB	LC-MS/MS(TMT/SRM/MRM)/immunohistochemical	Bladder Cancer	[156]
Urine	EHD4↑, EPS8L1↑, EPS8L2↑, GBP3↑, GsGTPa↑, GTPase Nras↑, MUC4↑, RAI3↑, Resistin↑	Ultracentrifugation/WB	LC-MS/MS/WB	Bladder Cancer	[157]
Urine	APOA1↑, TTR↑, PIGR↑, HPX↑, AZGP1↑, CP↑	Differential ultracentrifugation/protein concentration	LC-MS/MS (DDA)/WB	Chronic Active Antibody-Mediated Rejection	[158]
Urine	Calbindin↑, SNAP23↑	Ultracentrifugation/NTA, TEM, WB	LC-MS/MS/WB	Parkinson’s Disease	[159]
Urine	AGP1↑	Differential ultracentrifugation/TEM, NTA, WB	LC-MS/MS/WB	Primary Aldosteronism	[160]
Urine	AQP1↓, CAIX↑, CD10↓, CD147↓, CP↑, DKK4↑, DPEP1↓, MMP9↑, PODXL↑, Syntenin-1↓	Differential centrifugation, density gradient ultracentrifugation, ultrafiltration/TEM, WB, NTA	LC-MS/MS/WB	Renal Cancer	[161]
Saliva	BASP1↑, NUCB2↑, PSMA7↑, PSMB7↑, TKT↑, TLN1↑, WDR1↑	Centrifugation, exosome isolation kit/EM, WB	LC-MS/MS/WB	Inflammatory Bowel Disease/Ulcerative Colitis/Crohn’s Disease	[162]
Tear and Saliva	STOM↑, ANXA4↑, ANXA1↑	Size-exclusion chromatography/NTA, flow cytometry	LC-MS/MS/NA	Primary Sjögren’s Syndrome	[163]

NTA, nanoparticle tracking analysis; WB, western blot; TEM, transmission electron microscope; SEM, scanning electron microscope; DLS, dynamic light scattering; ELISA, enzyme-linked immunosorbent assay; LVSEM, low-vacuum scanning electron microscope; TMT, tandem mass tag; DDA, data-dependent acquisition; SRM, selective reaction monitoring; MRM, multiple reaction monitoring; PRM, parallel reaction monitoring; LC-MS/MS, liquid chromatography with tandem mass spectrometry; UPLC, ultra-performance liquid chromatography; MALDI-TOF-MS, Matrix-Assisted Laser Desorption/Ionization Time-of-Flight mass-spectrometer; NA, not available; ↑, upregulated; ↓, downregulated.

**Table 6:** List of potential disease biomarkers derived from EVs in human body fluids via transcriptome studies.

Source	Type	Biomarker	Isolation Method/Identification Method	Screening Method/Verification Method	Disease	Ref.
Serum	circular RNAs	Chr10q11↑, Chr1p11↑, Chr7q11↑	exoRNeasy Midi kit, ultracentrifugation/TEM, NTA, WB	RNA Seq/RT-qPCR	Gastric Cancer	[164]
Plasma		circRNAs↑	Differential centrifugation/cryo-EM, NTA	RNA-Seq/NA	Multiple Sclerosis	[165]
Serum	long non-coding RNAs	HULC↑	Ultracentrifugation/-	Microarray/RT-qPCR	Pancreatic Cancer	[166]
Serum		LINC00853↑	ExoQuick/TEM, NTA, WB	RNA Seq/RT-qPCR	Hepatocellular Carcinoma	[167]
Plasma		RP3-399L15.2↓, CH507-513H4.6↓	exoRNeasy/TEM, NTA, WB	RNA Seq/RT-qPCR	Endometriosis	[168]
Plasma	exLR	NFKBIA↑, NDUFB10↑, SLC7A7↑, ARPC5↑, SEPTIN9↑, etc.	Ultracentrifugation/TEM, NTA, WB	RNA Seq/RT-qPCR	Lung Cancer	[169]
Plasma	microRNAs	hsa-miR-106b-3p↑, hsa-miR-125a-5p↑, hsa-miR-3615↑, et al.	Ultracentrifugation/TEM, NTA, WB	RNA Seq/RT-qPCR	Lung Cancer	[170]
Plasma		hsa-miR-186-5p↑, hsa-miR-200c-3p↑, hsa-miR-429↑, etc.	SEC/TEM, NTA, WB	RNA Seq/RT-qPCR	Gastric Cancer	[171]
Plasma	long RNAs	hsa-miR-483-5p↑	Total Exosome Isolation Kit, differential ultracentrifugation/TEM, DLS, flowcytometry	Microarray/RT-qPCR	Adrenocortical Tumors	[172]
Plasma	microRNAs	microRNA-29a↑	Differential centrifugation, density gradient centrifugation/TEM, NTA, WB	RNA Seq/ddPCR	Chronic Methamphetamine Use Disorder	[173]
Serum		MicroRNA-431-5p↑	Differential centrifugation/TEM, NTA, WB	Microarray/RT-qPCR	Diabetic Retinopathy	[174]
Plasma		microRNA-491-5p↑	ExoQuick/TEM, NTA, WB	NanoString miRNAs analysis/RT-qPCR	Head and Neck Squamous Cell Carcinoma	[175]
Plasma		miR-101↓	Differential centrifugation/TEM, NTA, WB	RNA Seq/RT-qPCR	Osteosarcoma	[176]
Plasma		miR-101-3p↓, miR-150-5p↑	Precipitation/TEM, NTA, WB, ExoView	RNA Seq/RT-qPCR	Lung Cancer	[177]
Plasma		miR-103a-3p↑, miR-30e-3p↓	Ultracentrifugation/TEM, NTA, flow cytometry	OpenArray/RT-qPCR	Malignant Pleural Mesothelioma	[178]
Serum		miR-122-5p↑, miR-2110↑, miR-483-5p↑; miR-370-3p↓, miR-409-3p↓, etc.	miRCURY/NA	RNA-Seq/RT-qPCR-	Atherosclerosis	[179]
Serum		miR-1246↑	SEC/TEM, NTA	Microarray/RT-qPCR	Gallbladder Cancer	[180]
Plasma		miR-127-3p↓, miR-155-5p↓, miR-21-5p↓, miR-24-3p↓, let-7a-5p↓	SEC/NA	RNA Seq/RT-qPCR	Classical Hodgkin Lymphoma	[181]
Plasma		miR-134-5p↓, miR-205-5p↑, miR-409-3p↓	SEC/TEM, NTA, WB	RNA Seq/RT-qPCR	Nasopharyngeal Carcinoma	[182]
Plasma		miR-181a↑, miR-1908↑, miR-21↑, miR-486↑, miR-223↑	ExoQuick, exoRNeasy/NA	RNA Seq/NA	Ovarian Cancer	[183]
Serum		miR-181a-5p↑	Total exosome isolation kit/TEM, NTA, WB	Microarray/RT-qPCR	Prostate Cancer	[184]
Serum		miR-21-5p’(3′ addition C)↑, miR-23a-3p↑, tRF-Lys↑	Total exosome isolation kit/TEM, NTA, WB	RNA Seq/NA	Breast Cancer	[185]
Serum		miR-223↑, let-7e-5p↑, miR-486-3p↑, etc.	ExoQuick/TEM, NTA, flowcytometry	RNA Seq/RT-qPCR	Acute Rejection	[186]
Plasma		miR-22-3p↑, miR-99a-5p↑, miR-151a-5p↑, miR-320b↑, miR-320d↑, etc.	ExoQuick, Exo-Spin/TEM, NTA, tunable resistive pulse sensing, WB	RNA Seq/RT-qPCR	Chronic Obstructive Pulmonary Disease	[187]
Serum		miR-342-3p↑, miR-1254↓	ExoChip/SEM, NTA, WB	NanoString miRNAs Analysis/NA	Sporadic Amyotrophic Lateral Sclerosis	[100]
Plasma		miR-92b-3p↑, miR-374a-5p↑, miR-106b-3p↑	miRCURY/NTA, TEM, WB	RNA Seq/RT-qPCR	Chronic Obstructive Pulmonary Disease	[188]
Plasma		miRNA-152-3p↑, miRNA-1277-5p↑	SEC/NTA, TEM, WB	RNA Seq/RT-qPCR	Lung Cancer	[189]
Serum		miRNA-21↑	ExoQuick/NTA, WB	miRNA array/RT-qPCR	Chronic Lung Disease	[190]
Plasma		miRNAs, miR-500a-3p↑, miR-501-3p↑, miR-502-3p↑	3D medicine isolation reagent, polyethylene glycol-based method/NTA, SEM, WB	RNA Seq/NA	Pulmonary Ground-Glass Nodules	[191]
Plasma		Let-7b-5p↑, miR-184↓, circulating miR-22-3p↓	SEC/NTA, EM, WB	RNA Seq/RT-qPCR	Lung Cancer	[192]
Plasma		let-7e↑	Norgen plasma, serum exosome purification mini kit/WB	RNA Seq/RT-qPCR	Alzheimer’s Disease	[193]
Plasma		let-7i-5p↑	ExoQuick/TEM, NanoFCM, WB	RNA Seq/RT-qPCR	Asthma	[194]
Serum	piRNAs	DQ593039↑	Total exosome isolation reagent, exoEasy kit/TEM, NTA, WB	RNA Seq/RT-qPCR	Pulmonary Hypertension	[195]
CSF	microRNAs	miR-21↑	miRCURY/TEM, NanoFCM, WB	RNA Seq/ddPCR	Leptomeningeal Metastasis	[196]
Urine	microRNAs	hsa-miR-193b-3p↓, hsa-miR-8485↓	miRCURY/ExoView	miRNA Seq/NA	Acute Exercise-Induced Fatigue	[197]
Neurosurgical aspirate fluids	microRNAs	miR-486-3p↑	Ultracentrifugation/TEM, NTA, WB	RNA Seq/NA	Glioblastoma	[198]
TDV	microRNAs	miR-203a-3p↑	Ultracentrifugation/TEM, NTA, WB	RNA Seq/RT-qPCR	Lung Cancer	[199]

TDV, tumor-draining vein; CSF, cerebrospinal fluid; NTA, nanoparticle tracking analysis; WB, western blot; TEM, transmission electron microscope; SEM, scanning electron microscope; Cryo-EM, cryoelectron microscopy; DLS, dynamic light scattering; exLR, extracellular vesicle long RNA; piRNAs, P-element induced wimpy testis (PIWI)-interacting RNAs; ddPCR, droplet digital polymerase chain reaction; NA, not available; ↑, upregulated; ↓, downregulated.

## 6. The Identification of EVs

For new benchmarks in the characterization of EVs, almost all the literature still refers to the MISEV2018 (a consensus of ISEV) [12]. It is necessary to identify EVs and aim to ensure the biological functions of EVs merely. Multiple and complementary methods are used to assess the purity, morphology, and quantification of EVs, such as the reviewed above identification methods in Table 5 and Table 6, western blot, NTA, and Cryo-EM are often used to characterize EVs for downstream study. Recently, ExoView [121], a nanoflow cytometry instrument that combines immunoaffinity with high-resolution imaging techniques for specific exosomes, was developed and led to a more convenient and streamlined routine for characterizing the count, size (>50 nm), and surface markers of EVs without sample purification to capture EVs with antibodies, then measure surface protein expression levels via fluorescent signals to evaluate multiple metrics on the individual particle at a high-throughput level. For EV proteomic research, EV identification is a part of the workflow. An efficient, quick, automated, and robust standard characterization process also is essential for subsequent unbiased analysis of the physiology and pathology of the disease.

## 7. The Proteomic Profile Workflow of EVs in Clinical Investigation

**Figure 4:**
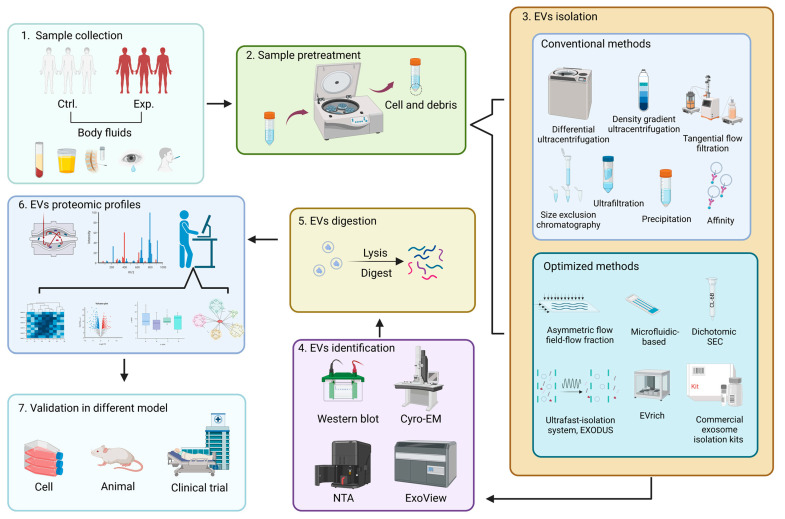
The workflow of collecting EVs of clinical biofluid for proteomics analysis (created with BioRender.com).

In general, there are two proteome analyses that are bottom-up and top-down, respectively; the former is popular in many labs since it has fewer instrument and software requirements and is more established than the latter one. For researchers interested in setting up their own workflow, published experimental protocols using different approaches and targeting different aspects of the proteome [200,201,202,203] are a good starting point. A generic bottom-up workflow is depicted in Figure 4 and consists of the following steps: 1. Sample collection from clinical biofluid with appropriate storing and processing conditions; 2. Low-speed centrifugation to remove cells and debris as sample pretreatment procedure; 3. Followed by conventional or optimized methods to purify EVs, further (optional) purification to obtain more homogenous EVs via combining other methods; 4. Then characterization of EVs to ensure the purity conforms to the experimental requirements; 5. For lysis and digestion of EVs enriched in MS-compatible buffers, FASP is widely adopted for single-shot, label-free or label-based (TMT and iTRAQ) LC-MS/MS analysis; 6. Identification, quantification, and statistical analysis of data by MaxQuant, Perseus, and Proteome Discoverer software, etc.; 7. Finally, validation in a suitable model to experimentally assess the value of certain proteins for intended future application (e.g., as a biomarker).

## 8. Challenges for Proteomics of EVs in Clinical Investigation

There are three crucial questions for EVs: Where are they from? Where are they going? What do they do? Answering the first question is impeded by available methods because it is hard to obtain a pure EVs subpopulation from biofluid by centrifugation by applying existing protocols due to the size of other nanoparticles and inevitable contamination that affects the proteomic profiles. Next, the isolation of EVs from a different sample such as plasma, the most widely used in disease research, is more demanding than urine and cerebrospinal fluid given the presence of large protein aggregates, chylomicrons, protein–nucleic acid aggregates, and plasma proteins. In addition, importantly, the EV population derived from biofluid is heterogeneous and presents an extraordinarily complicated mixture of host and disease-related particles, hence the paramount challenge of extracting EVs from specific cells. Moreover, it is difficult to separate homogenous EV populations due to the diversity of the molecular distribution of EVs, with only a few particles identical to each other even when released from a single cell type. In addition, there is an intrinsic and large inter-individual variability between clinical samples. Additionally, due to the proteomic profiles being highly dependent on the isolation process of EVs, as mentioned before [90,92,93,94], a mostly automated and traceable workflow for biofluid handling and analysis is essential for quality control and analysis of the proteome data.

## 9. Recent Progress and Future Directions in EV Proteomics

While ELISA is the method of choice for high-throughput, sensitive, quantitative, and qualitative analysis of known EV protein biomarkers, mass spectrometry discovers new biomarkers and promising protein signatures specific to different diseases. Manifold development (Table 7), including the isolation and characterization of EVs, the PTM enrichment strategy of EVs, minimizing tradeoffs between throughput and depth of MS in unbiased analysis, multi-omics techniques for molecular panels, signaling pathways, and pharmacokinetics of EVs in particular pathological status, and final validation in biological effects of key protein from EVs, have been promoted by scientists in proteomics profiles of EVs since a review by Rocha et al. [204] in 2017. The heterogeneity between and within EVs is still challenging research to distinguish and understand the role of EVs in complicated body fluids. Fortunately, the nucleic acids can be amplified to obtain clues for the heterogeneity of EVs; Ruben et al. [205] found EXOmotifs and CELLmotifs of miRNA-mediated sorting or retention in EVs, which provided important insight into the partial miRNA delivery system of EVs. However, it is impractical to carry out MS for a single EV due to its tiny volume and the detection limit of current MS-based proteomics methods. Consequently, Wu et al. [206] developed a proximity-dependent barcoding assay to distinguish variability and the respective number of individual exosome surface proteins using antibody-DNA conjugates and next-generation sequencing. Another advanced single EV method designed by Ko et al. [207] utilized the single EV immune sequencing technology on a microfluidic-based droplet generator to enclose and link bead-derived DNA barcodes to complexes containing individual antibodies and EVs. Recently, Banijamali et al. [208] facilitated the verification of EV subtypes within and between samples via a scalable and relatively simple droplet barcode sequencing for surface protein analysis at a single EV level. For the isolation procedure of EVs, many developed methods [97,103,104,105,110] are applied to improve EV purity effectively and to reduce the long processing times; it remains important to screen for and report the most appropriate workflow of multiple integrated strategies for each specific research scenario and sample type. For the characterization of EVs, the widely used and classical approaches remain as western blot, NTA, and cryo-EM; here, there is progress seen in more accurate devices introduced to detect EVs’ multiple parameters simultaneously, such as high-resolution imaging nanoflow cytometry developed by Choi et al. [209] and hollow-fiber flow field-flow fractionation (HF5) designed by Marassi et al. [210]. As reported [211,212], shotgun proteomics for discovery studies is the most popular technique to analyze the proteins of EVs; there are emerging techniques, such as targeted quantification with selected/multiple/parallel reaction monitoring (SRM/MRM/PRM) and DIA acquisition, such as sequential window acquisition of all theoretical mass spectra (SWATH-MS), all greatly facilitating proteome coverage capabilities, efficient validation, and accuracy. For the PTM-proteome of EVs, Andaluz Aguilar et al. [203] described a workflow for the sequential analysis of phosphopeptides, N-glycopeptides, and total proteome analysis from 1 mL of human plasma showcasing the high sensitivity and high enrichment efficiency of current instruments and methods for low-abundance PTM-proteome. As mentioned, proteome profiles are highly dependent on the whole workflow; to ensure unbiased processing and reduce technical variance, Ozge et al. [213] integrated an automated and high-throughput sample preparation (Agilent Bravo© liquid handling platform) in proteomics to detect Parkinson’s disease and achieve patient stratification for precision therapy. Multi-omics boosts our systems biology understanding of the EV function via obtaining rich molecular information because a single omics study cannot reveal the complexities of a living system entirely. Cohn et al. [65] revealed disease-associated signatures of EVs from human AD brain tissue via proteomics, lipidomics, and miRNA transcriptomics. Noteworthily, Heo et al. [113] reviewed multi-omics approaches in cancer research and pointed out the computational and biological challenges. The final validation of biological effects after proteomic profiling for EVs is hampered by insufficient standardization between research labs, which hinders the clinical translation in diagnostic and therapeutic applications, emphasizing the need for further establishment of routine workflows by ongoing research and collaborative efforts from many different fields.

## 10. Conclusions

Proteomics has largely contributed to our understanding of EVs, which have emerged as a promising tool for clinical diagnosis, prognosis, and therapeutic interventions. The whole field of proteomic research of EVs in clinical biofluids, such as blood, urine, and cerebrospinal fluid, has matured in recent years where numerous works contributed to the establishment of experimental tools and guidelines. However, limitations and challenges have been identified, which must be addressed in the future: For research, these lie mainly in further improving the purification and characterization of EVs to unravel evermore biological details. For clinical applications, further improvement of standardization, comparability, and reproducibility are key to wider acceptance and use. We can expect for the future a prominent role of proteomics for EVs in clinical biofluids in advancing personalized medicine and improving patient outcomes.

## Figures and Tables

**Table 1:** Characteristics of EVs.

Feature	Exosome	Microvesicle	Apoptotic Body
Size (nm)	40–150	150–1000	1000–5000
Density (g/mL)	1.13–1.19	1.25–1.30	1.16–1.28
Origin	Living cell	Living cell	Dying cell
Process	Releasing ILVs during plasma membrane fusion of MVBs	Budding from the plasma membrane directly	Blebbing from the plasma membrane during cell apoptosis
Contents	Nucleic acid, protein, lipid, etc.	Nucleic acid, protein, lipid, etc.	Fragments of the cellular components
Markers [28]	CD63, TSG101, Alix, HSP70, etc.	Integrins, selections, CD40	Histones, TSP, C3b
Clinical application	Diagnosis, therapy [1,29]	Diagnosis, therapy [27,30]	Emerging [31]
Biomarker and therapeutic research ^	High [32]	Medium [15]	Low [31,33]

^: research quantity.

**Table 2:** Characteristics of EVs enrichment strategies.

Method	Yield	Purity	Time	Workload	Price	Practice
*Conventional approaches*						
Differential ultracentrifugation	High	Medium	Long	>100 mL	Low	Easy
Ultrafiltration	High	Low	Medium	>100 mL	Medium	Easy
Tangential flow filtration	High	Medium	Medium	>100 mL	Medium	Medium
Size-exclusion chromatography	Low	Medium	Short	Up to a few mL	Medium	Easy
Density gradient ultracentrifugation	Low	High	Long	Up to 1 mL	Medium	Medium
Precipitation	High	Low	Medium	>100 µL	Low	Easy
Affinity	Low	High	Long	Up to 1 mL	High	Medium
*Advanced approaches*						
AF4	Low	High	Medium	100 µL	/ ^#^	Medium
Microfluidic-based technologies	Low	High	- *	>10 µL	High	Medium
Dichotomic SEC	Medium	High	Short	20 mL	Medium	Easy
EXODUS	High	High	Short	>100 mL	Medium	Easy
EVrich	- *	High	- *	/ ^#^	/ ^#^	Easy
Commercial EV isolation kits	High	High	Various	Various	High	Easy

*: A streamlined workflow platform for downstream analysis; ^#^: Unknown.

**Table 3:** List of commercial exosome isolation kits and separation principles.

Commercial Exosomes Isolation Kits	Separation Principle	Company	Cat. No.
Capturem	Affinity, lectin	Takara	635741
EasySep	Affinity, antibody	Stem Cell	100-0812
exoEasy	Affinity, membrane-based	Qiagen	76064
ExoPure	Precipitation	Abcam	ab287883
ExoQuick	Precipitation	System Biosciences	EXOQ20A-1
Exo-spin	Size-exclusion chromatography	Cell Guidance Systems	EX05
ExoSure	Size-exclusion chromatography	Gene Copoeia	EP001
Hieff	Precipitation	YEASEN	41201ES25
MagCapture	Affinity, phosphatidylserine	FUJIFILM Wako	290-84103
miRCURY	Precipitation	Qiagen	76603
PureExo	Precipitation	101Bio	P101
Total Exosome Isolation Reagent	Precipitation	Invitrogen	4478359

**Table 4:** Common -omics research in EVs.

Omics	Subject	Current Challenges
Genomics	DNA [116]	The DNA of EVs is still difficult to preserve and isolate [117].
Epigenomics	DNA [118]	The interpretation of the data from dynamic and specific tissue [119].
Transcriptomics	RNA	A relatively robust method has been studied in high throughput even at a single EV level [120]. Now, analysis is the bottleneck.
Proteomics	Protein	Comprehensive, reproducible, and accurate data depends on the purity of EVs [121]; sensitivity and throughput.
Metabolomics/Lipidomics/Glycomics	Metabolite [122]/Lipid [123]/Glycan [124]	The sensitivity, reproducibility, robustness, speed, and accuracy; the investigation of EV subpopulation; comprehensive analysis.

**Table 7:** Challenges and future directions in EVs proteomics.

Challenges	Future Directions
1. The heterogeneity of EVs.	Applying integrated module approaches at a single EV level.
2. The purity of EVs.	Selecting the most appropriate approach for each specific research scenario and sample type.
3. The identification methods of EVs.	Considering reproducible and accurate approaches.
4. The proteome coverage of EVs by LC/ESI-MS/MS and DDA/DIA.	Alternative data analysis strategies according to set parameters with robust bioinformatic tools.
5. The PTM-proteome of EVs.	High capture efficiency of enrichment strategy.
6. Tradeoffs between throughput and depth of LC-MS/MS.	Robust, automated, and high-throughput workflow.
7. Systems biology overview of the EVs function.	Advanced computational and biological algorithms.
8. Final biological effects validation in EVs.	A routine research workflow between labs and collaborative efforts from many different fields.

## Data Availability

No new data were created or analyzed in this study. Data sharing is not applicable to this article.
